# Surface ocean pH and buffer capacity: past, present and future

**DOI:** 10.1038/s41598-019-55039-4

**Published:** 2019-12-09

**Authors:** Li-Qing Jiang, Brendan R. Carter, Richard A. Feely, Siv K. Lauvset, Are Olsen

**Affiliations:** 1 Earth System Science Interdisciplinary Center, University of Maryland, College Park, Maryland USA; 2 National Centers for Environmental Information, National Oceanic and Atmospheric Administration, Silver Spring, Maryland USA; 3 Joint Institute for the Study of the Atmosphere and Ocean, University of Washington, Seattle, Washington, USA; 4 Pacific Marine Environmental Laboratory, National Oceanic and Atmospheric Administration, Seattle, Washington, USA; 5 NORCE Norwegian Research Centre, Bjerknes Centre for Climate Research, Bergen, Norway; 6 Geophysical Institute, University of Bergen and Bjerknes Centre for Climate Research, Bergen, Norway

**Keywords:** Carbon cycle, Marine chemistry

## Abstract

The ocean’s chemistry is changing due to the uptake of anthropogenic carbon dioxide (CO_2_). This phenomenon, commonly referred to as “Ocean Acidification”, is endangering coral reefs and the broader marine ecosystems. In this study, we combine a recent observational seawater CO_2_ data product, i.e., the 6^th^ version of the Surface Ocean CO_2_ Atlas (1991–2018, ~23 million observations), with temporal trends at individual locations of the global ocean from a robust Earth System Model to provide a high-resolution regionally varying view of global surface ocean pH and the Revelle Factor. The climatology extends from the pre-Industrial era (1750 C.E.) to the end of this century under historical atmospheric CO_2_ concentrations (pre-2005) and the Representative Concentrations Pathways (post-2005) of the Intergovernmental Panel on Climate Change (IPCC)’s 5^th^ Assessment Report. By linking the modeled pH trends to the observed modern pH distribution, the climatology benefits from recent improvements in both model design and observational data coverage, and is likely to provide improved regional OA trajectories than the model output could alone, therefore, will help guide the regional OA adaptation strategies. We show that air-sea CO_2_ disequilibrium is the dominant mode of spatial variability for surface pH, and discuss why pH and calcium carbonate mineral saturation states, two important metrics for OA, show contrasting spatial variability.

## Introduction

Ocean pH, carbonate ion concentrations ([CO_3_^2−^]), and calcium carbonate mineral saturation states (Ω) have been declining as a result of the uptake of approximately 30% of the anthropogenic carbon dioxide (CO_2_) emissions over the past 270 years^1–6^. This process is commonly referred to as “ocean acidification (OA)”^1,2,7,8^. As the “other CO_2_ problem”, OA is making it harder for marine calcifiers to build a shell and/or skeletal structure, endangering coral reefs and the broader marine ecosystems^1,2,9–11^.

Seawater pH measurements date back to the beginning of the 20^th^ century^12^, yet very few historical pH data are adequate for studying the global pH distribution. Prior to 1989, seawater pH was typically measured using glass electrodes with uncertainties as much as 0.1 units. Moreover, the earlier marine pH records are often unclear about the pH scale, measurement temperature, and whether the reported pH has been adjusted from measurement to *in-situ* conditions^13^. Only since the late 1980s when spectrophotometric pH measurement methods were refined, has it become possible for labs using dye solutions to discern small pH changes such as those expected from OA^14–16^. Additionally, the accepted spectrophotometric seawater pH measurement practices have been recently updated to allow for corrections for the influences of dye impurities^17,18^_,_ challenging the comparability of pH measurements made between decades.

In lieu of measured pH, global studies of seawater pH have been relying on values calculated from other seawater CO_2_ chemistry variables. The modern day surface ocean pH distribution was recently described by Takahashi *et al*.^19^ who calculated pH (adjusted to 2005) using a gridded partial pressure of carbon dioxide (*p*CO_2_) data product from the Lamont Doherty Earth Observatory (utilizing ~6 million observations), and total alkalinity (TA) estimated from gridded sea surface salinity and nitrate. The historical and future pH distributions can be directly extracted from Earth System Models^20,21^, but the surface ocean pH distributions in models are controlled by the modeled processes and do not always closely reflect the true ocean state^21^. Furthermore, the model initial conditions are usually based on the first version of the Global Ocean Data Analysis Project (GLODAP) data product (1972–1999, ~6000 observations) with limited spatial coverage^22^.

Following the approach of Orr *et al*.^2^, we combine a recent rich observational CO_2_ data product, i.e., the 6^th^ version of the Surface Ocean CO_2_ Atlas (SOCATv6, 1991–2018, ~23 million observations)^23^, with an updated TA estimation routine (locally interpolated alkalinity regression, or LIARv2)^24^, and temporal trends at individual locations of the global ocean from a robust Earth System Model (ESM2M)^20^ to provide a high-resolution regionally varying view of global surface ocean pH and Revelle Factor^25^ from the pre-Industrial era (1750 C.E.) to the end of the century.

Revelle Factor is a measure of the ocean’s buffer capacity for the carbonate system in seawater or freshwater. It is defined as the ratio between the fractional change in *p*CO_2_ to the fractional change in dissolved inorganic carbon (DIC) (Eq. 1) at constant temperature, salinity and TA^25^.

1 $${\rm{Revelle}}\,{\rm{factor}}=(\Delta p{{\rm{CO}}}_{2}/p{{\rm{CO}}}_{2})/(\Delta \text{DIC}/{\rm{DIC}})$$

The higher the Revelle factor, the lower the ocean’s buffer capacity, and the faster the change of *p*CO_2_ in the ocean at a given DIC change. The Revelle Factor also serves as a good indicator of the ocean’s buffer capacity in terms of pH changes. A similar quantity defined as the ratio between the fractional change in pH to the fractional change in DIC using our data shows a strong correlation with the Revelle Factor (R^2^ > 0.999), so we discuss variability in these two related factors as though they are interchangeable. In this study, we report on the spatial and temporal variations of surface pH and Revelle Factor, their seasonal variability, and long-term changes from 1770 to 2100.

### Surface pH distribution

**Figure 1:**
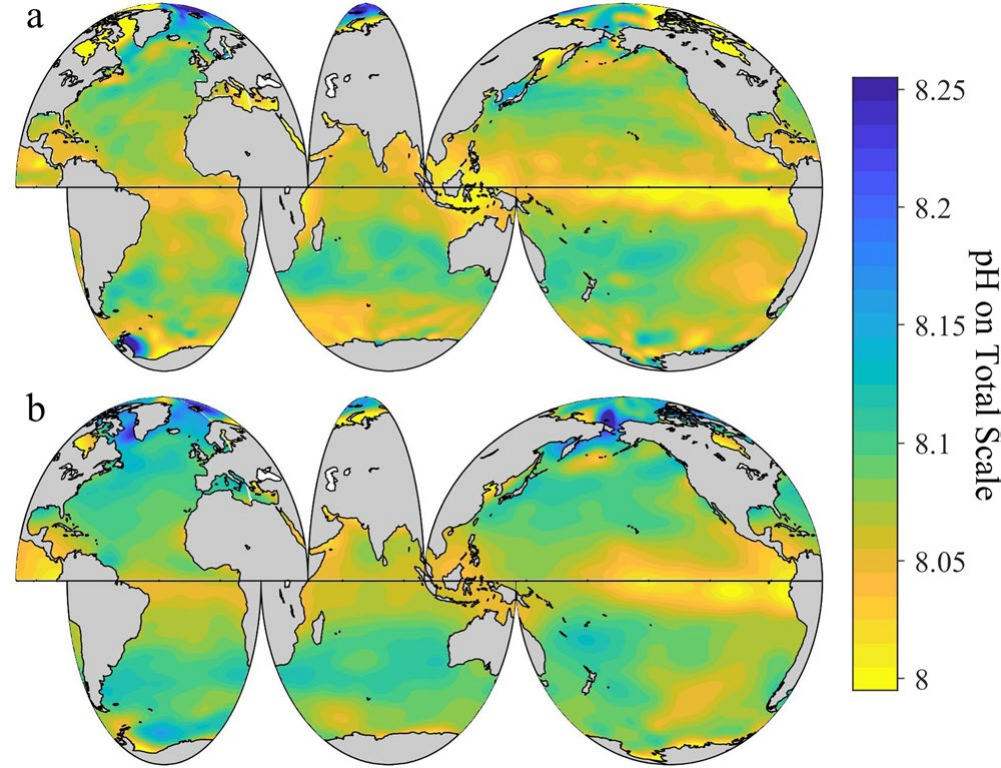
Climatological distribution of global surface ocean pH on the total hydrogen scale (pH_T_) at *in-situ* temperature. The pH_T_ values are annually averaged and adjusted to be approximate for the year 2000. Panel a, surface pH_T_ based on the 6^th^ version of the Surface Ocean CO_2_ Atlas (SOCATv6). Panel b, surface pH_T_ based on the 2^nd^ version of the Global Ocean Data Analysis Project (GLODAPv2).

The pH results of this study calculated using the SOCATv6 data product are statistically indistinguishable (mean ± root-mean-squared difference: 0.005 ± 0.014) from those calculated from the 2^nd^ version of Global Ocean Data Analysis Project data product (GLODAPv2)^26^ (Fig. 1 and Supplementary Fig. S1). However, due to the much better spatial coverage (23 million vs. 15,115 observations) and consummately lower gridding uncertainty, and to a lesser extent the smaller pH uncertainties (0.01 vs. 0.02 pH) of the SOCATv6 calculations than the GLODAPv2 calculations, we contend that the SOCATv6-based gridded values are preferable to the GLODAPv2-based gridded values at the ocean surface. Except where otherwise indicated, the results of this study are therefore based on SOCATv6.

The pH climatology we produce shows a similar (a negligible average difference of 2.6 × 10^−4^ pH units) pH to that directly extracted from the Earth System Model that we use to derive temporal trends, suggesting the model captures the important processes controlling average surface ocean pH well (Supplementary Fig. S2). However, the two pH distributions show large differences regionally (a root-mean-squared difference of 0.06 pH units at the surface, equivalent to a 15% acidity difference, or ~3 decades of pH change^27^). The hybrid climatology we produce, which combines observation-based distribution with model-based temporal trends at individual locations of the global ocean, is likely to provide improved regional OA trajectories than the model output could alone, and thus will help guide the regional OA adaptation strategies.

The annually-averaged global surface ocean pH_T_^2000^ (adjusted for the year 2000, gridded to a global 1 × 1° grid, see methods for details) between 60°N and 60°S shows a narrow range across the global surface ocean (<0.2 units, Fig. 1a). The lowest surface ocean pH_T_^2000^ is found in the equatorial regions from 20°N to 20°S, especially in the eastern Pacific. The Arctic Ocean shows the largest spatial variability, followed by the Southern Ocean. The globally and annually-averaged surface ocean pH_T_^2000^ in the Atlantic, Pacific, and Indian Oceans (60°N to 60°S) is 8.07 ± 0.02 (1σ uncertainty on gridded values, see Methods), 8.06 ± 0.03, and 8.07 ± 0.02, respectively, with a global average of 8.07 ± 0.02 between 60°N to 60°S.

### Two cancelling effects of SST on pH

pH and calcium carbonate mineral saturation states (Ω) are two important metrics for OA because they both respond sensitively to anthropogenic CO_2_ and are aspects of seawater chemistry that are thought to play a role in the health of some marine organisms. However, unlike surface ocean aragonite saturation state (Ω_arag_), which shows a large latitudinal gradient (e.g., Ω_arag_ varies from ~3.7 at sea surface temperature, or SST, of ~25 °C in the subtropical and tropical regions to ~1.4 at SST of ~0 °C in the high latitudes) and is strongly correlated with SST (R^2^ = 0.94)^28^, surface ocean pH_T_^2000^ shows a small latitudinal gradient (in the above example, pH only varies from 8.09 to 8.11) and is weakly correlated with SST (R^2^ = 0.056, Supplementary Fig. S3). Understanding this difference between pH and Ω requires exploring how temperature interacts with the two properties. Temperature controls surface ocean pH and Ω primarily through two processes: directly through the temperature dependence of the seawater CO_2_ chemistry; and indirectly through air-sea exchange of CO_2_ and the subsequent changes to the DIC/TA ratio of the water.

The first process controls the chemical speciation of CO_2_ dissolved in seawater. A simple model (see: Methods) shows that in a closed system with constant DIC and TA, when temperature increases from 20 to 25 °C, the dissociation of bicarbonate (HCO_3_^−^) and water (H_2_O) are the primary processes producing hydrogen ions (H^+^) (1.99 and 1.55 µmolkg^−^^1^, respectively, in this particular example, see: Methods), a large portion of which (2.72 µmolkg^−^^1^) is consumed by borate (B(OH)_4_^−^), with most of the remaining produced H^+^ (0.82 µmolkg^−1^) reacting with HCO_3_^–^ to form aqueous CO_2_ (CO_2_*, a combination of both dissolved CO_2_ and carbonic acid, or H_2_CO_3_), leaving a tiny portion of H^+^ (0.0015 µmolkg^−^^1^) in the seawater as H^+^ or H_3_O^+^, but enough to decrease pH from 8.10 to 8.02 (Supplementary Table S1). The extra CO_3_^2−^ from the dissociation of HCO_3_^–^ (an increase of ~2 µmolkg^−^^1^, or ~1%), combined with a decrease of apparent solubility product (K_sp_)with temperature (about –2% change from 20 to 25 °C)^29^ help enhance aragonite saturation state slightly (~3%) from 3.16 to 3.25.

The second process involves air-sea gas exchange and the associated changes in the DIC/TA ratio. When water temperature increases, the extra H^+^ from the dissociation of HCO_3_^−^ and H_2_O will react with the most abundant carbonic species in the ocean, HCO_3_^–^, driving it to build up excess aqueous CO_2_ (CO_2_*). In the above example, when temperature increases from 20 to 25 °C, [CO_2_*] increases by 0.82 µmolkg^−^^1^, which would translate to an *f*CO_2_ increase of ~25 µatm if CO_2_ solubility (K_0_) were not to change. In reality, K_0_ decreases with increasing temperature^30^, and the change of K_0_ alone would cause *f*CO_2_ to increase by an additional ~53 µatm, twice as much as that from the change of [CO_2_*]. In summary, when temperature increases, the changes of both [CO_2_*] and solubility (K_0_) work together to create a tendency for seawater to degas CO_2_ to the atmosphere, lowering the DIC/TA ratio^31^, thus raising both pH and aragonite saturation state. Similarly, colder temperature will enable a body of water to absorb more CO_2_ in order to maintain equilibrium with the atmosphere, thus increasing its DIC/TA ratio, and decreasing both pH and aragonite saturation state.

In the above example, if warming/cooling only impacted the seawater CO_2_ chemistry through speciation changes, surface pH_T_ would be 0.47 units higher near the poles than at the equator (Supplementary Fig. S4). In contrast, aragonite saturation state would be slightly (~15%) lower near the poles than at the equator. If the air-sea gas exchange of CO_2_ were the only controlling factor, pH_T_ in the polar area would be 0.46 units lower than in the tropical area, and aragonite saturation state would be ~50% lower. These effects nearly cancel for pH, whereas they reinforce each other for aragonite saturation state (Supplementary Fig. S4), which explains why surface ocean pH does not show as strong of a latitudinal gradient as aragonite saturation state (Supplementary Fig. S3).

### Role of air-sea CO_2_ disequilibria

**Figure 2:**
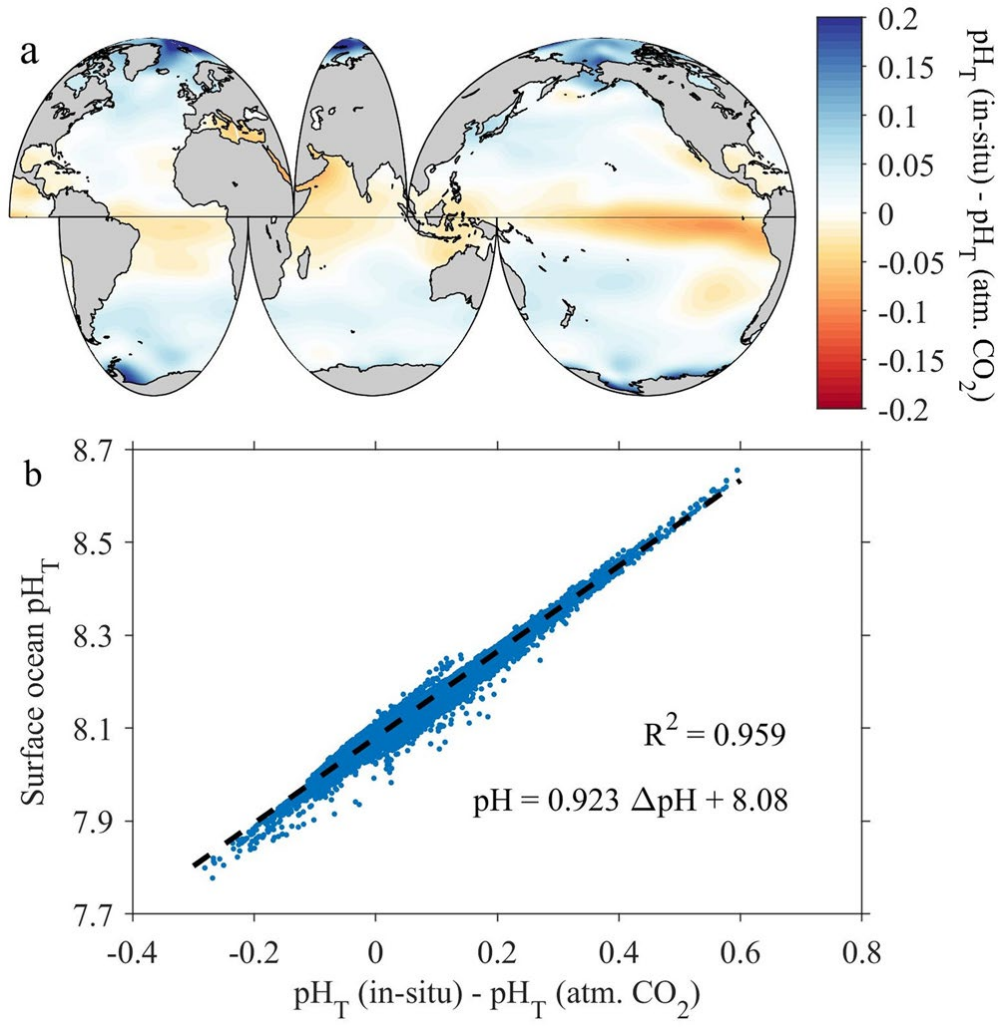
Surface ocean pH_T_ variation caused by air-sea CO_2_ disequilibria. Panel a, the spatial distribution of the pH caused by air-sea disequilibria. pH_T_ (atm. CO_2_) is the pH if the water were to be equilibrated with the atmospheric CO_2_ level in 2000. Panel b, the regression between surface pH_T_ and the pH caused by air-sea disequilibria based on the original discrete pH data (before gridding).

Air-sea gas exchange tends to bring surface ocean *p*CO_2_ close to equilibrium with the atmosphere, but disequilibria following temperature changes or biological drawdown of DIC or remineralization events do persist in the surface ocean^19^. We estimate the effect of air-sea CO_2_ disequilibria on surface ocean pH (ΔpH_T_^diseq^) from the differences between *in-situ* pH and the pH if the water were to be equilibrated with the atmospheric CO_2_. The results show that air-sea CO_2_ disequilibria elevate pH in high latitude areas where CO_2_ is undersaturated and decrease it in the equatorial upwelling region where CO_2_ is supersaturated (Fig. 2a)^19^. Globally, the average ΔpH_T_^diseq^ is 0.03 ± 0.06 (1σ). It should be noted that disequilibria are a symptom of pH variability rather than a cause, and the true explanation for any changes must require some combination of mixing, primary production, remineralization process, and heat exchange.

As discussed previously, the result of the two SST processes is a rather homogeneous surface ocean pH distribution from the tropical to the polar areas at equilibrium. To examine how strongly ΔpH_T_^diseq^ covaries with the observed surface ocean pH_T_ distribution, pH_T_ is plotted against ΔpH_T_^diseq^ (Fig. 2b). Their regression suggests that the spatial pH variability is strongly correlated with ΔpH_T_^diseq^ in the global surface ocean (R^2^ = 0.96, Fig. 2b). The surface air-sea disequilibria signal can be attributed to a time lag between the impacts of SST changes on seawater CO_2_ chemistry speciation which happen immediately, and those of the air-sea CO_2_ exchange which happen slowly following the temperature change.

### Pre-industrial surface ocean pH

**Figure 3:**
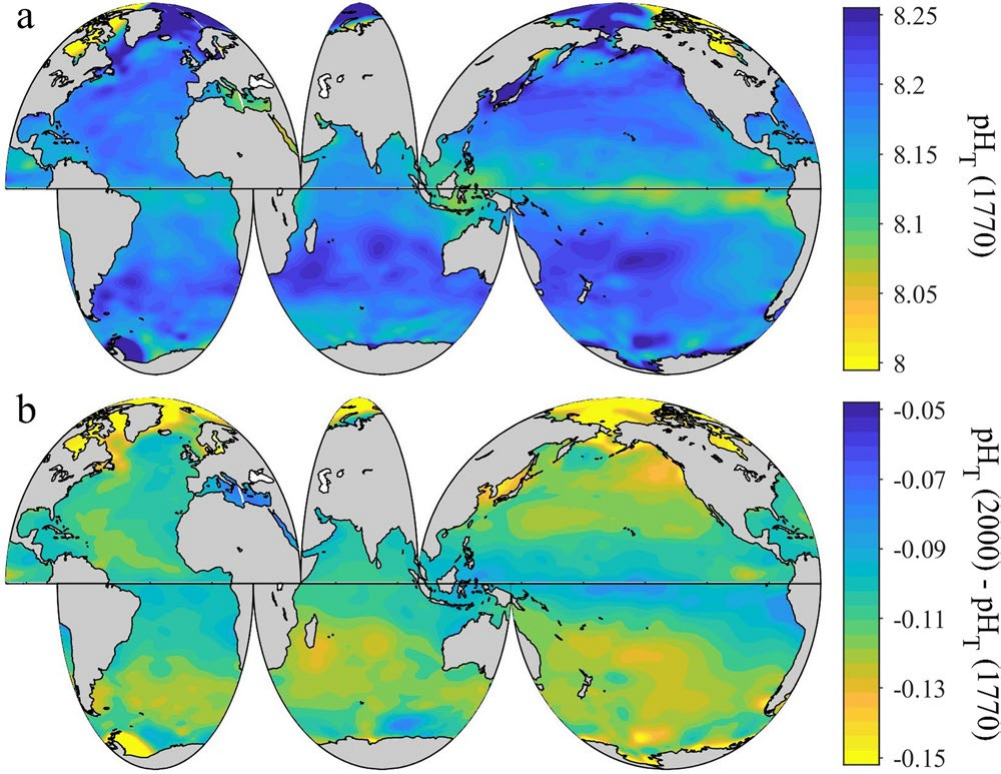
Spatial distribution of global surface ocean pH_T_ in 1770. Panel a, the annually-averaged surface ocean pH_T_ adjusted to be approximate for the year 1770. Panel b, the difference between pH_T_ in 2000 and 1770 (pH_2000_ – pH_1770_) in the global surface ocean.

**Figure 4:**
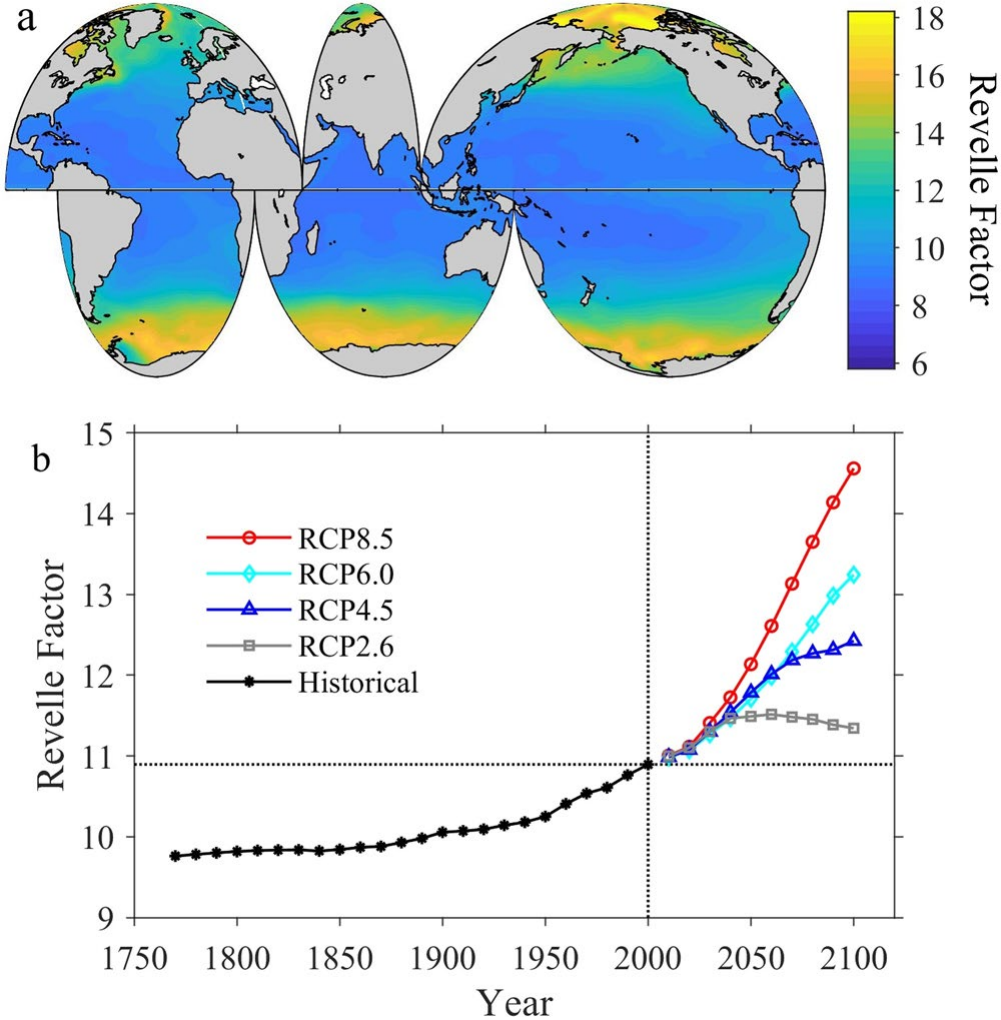
Spatial and temporal distributions of global surface ocean Revelle Factor. Panel a, the spatial distribution of annually-averaged Revelle Factor adjusted for the year 2000 in the global surface ocean. Panel b, the long-term change of the globally and annually-averaged surface Revelle Factor from 1770 to 2100 under all Representative Concentrations Pathway (RCP) scenarios.

Surface ocean pH_T_ in 1770 shows a similar spatial pattern as in 2000 (Fig. 3a), but is on average ~0.11 ± 0.03 (where the ± term reflects the root-mean-squared difference of this quantity across the global surface ocean) units higher in 1770 than in 2000 (Fig. 3b). Overall, the pH_T_ drop from 1770 to 2000 is fastest in areas with the greatest Revelle Factor (i.e., the lowest seawater buffer capacity) (Fig. 4a). The Arctic Ocean shows the greatest pH_T_ decrease (Fig. 3b), with an area-averaged ∆pH_T_ change of −0.16 ± 0.04 units from 1770 to 2000. In comparison, the equatorial region between 20°S and 20°N only shows an area-averaged ∆pH_T_ change of −0.10 ± 0.01 pH units during this period (Fig. 3b). The Southern Ocean data indicate strong area-averaged ∆pH_T_ declines, but not as severe as the declines in the Arctic Ocean.

### Future surface ocean pH

**Figure 5:**
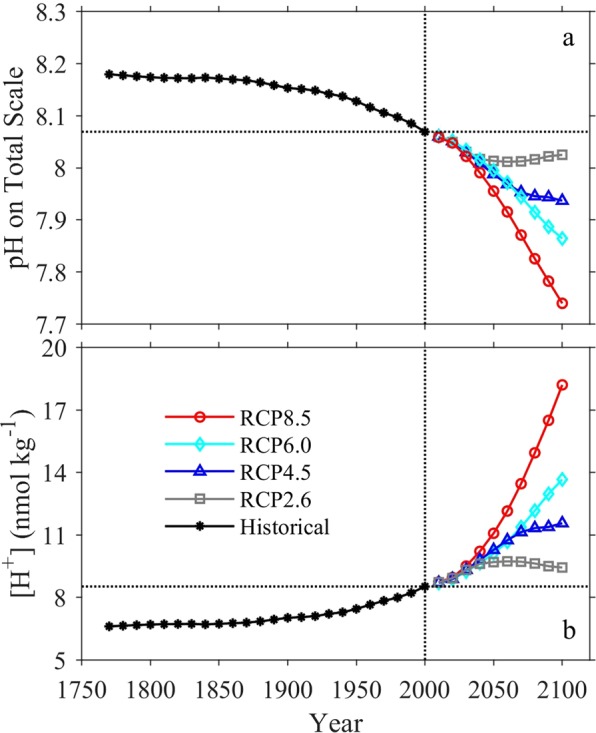
Long-term change of global surface ocean pH_T_ and ocean acidity. Panel a, the globally and annually-averaged surface ocean pH_T_ from 1770 to 2100. Panel b, the change of ocean acidity (hydrogen ion activity, [H^+^], 1 nmol kg^−1^ = 1×10^−9^ mol kg^−1^) over the same period.

**Figure 6:**
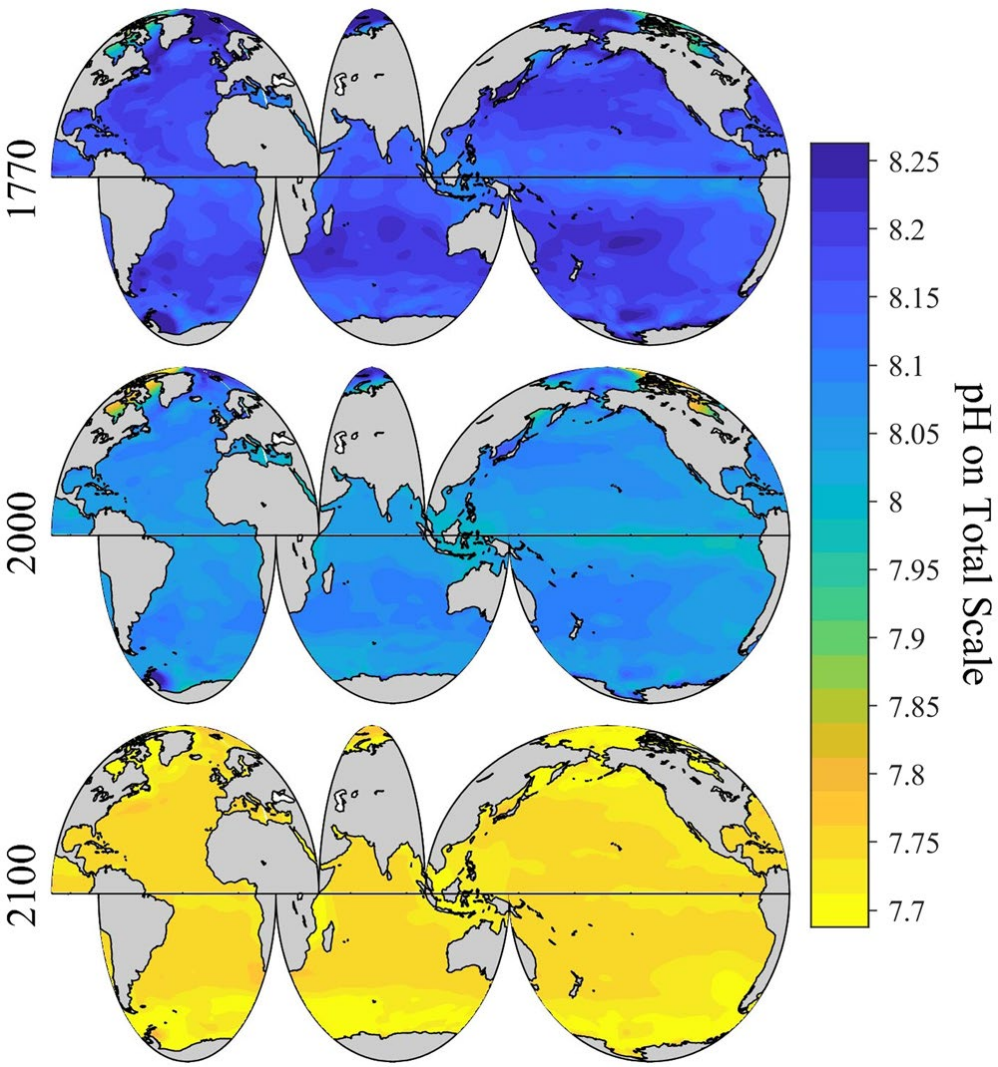
Spatial distribution of global surface ocean pH_T_ (total hydrogen scale, annually averaged) in past (1770), present (2000) and future (2100) under the IPCC RCP8.5 scenario.

**Figure 7:**
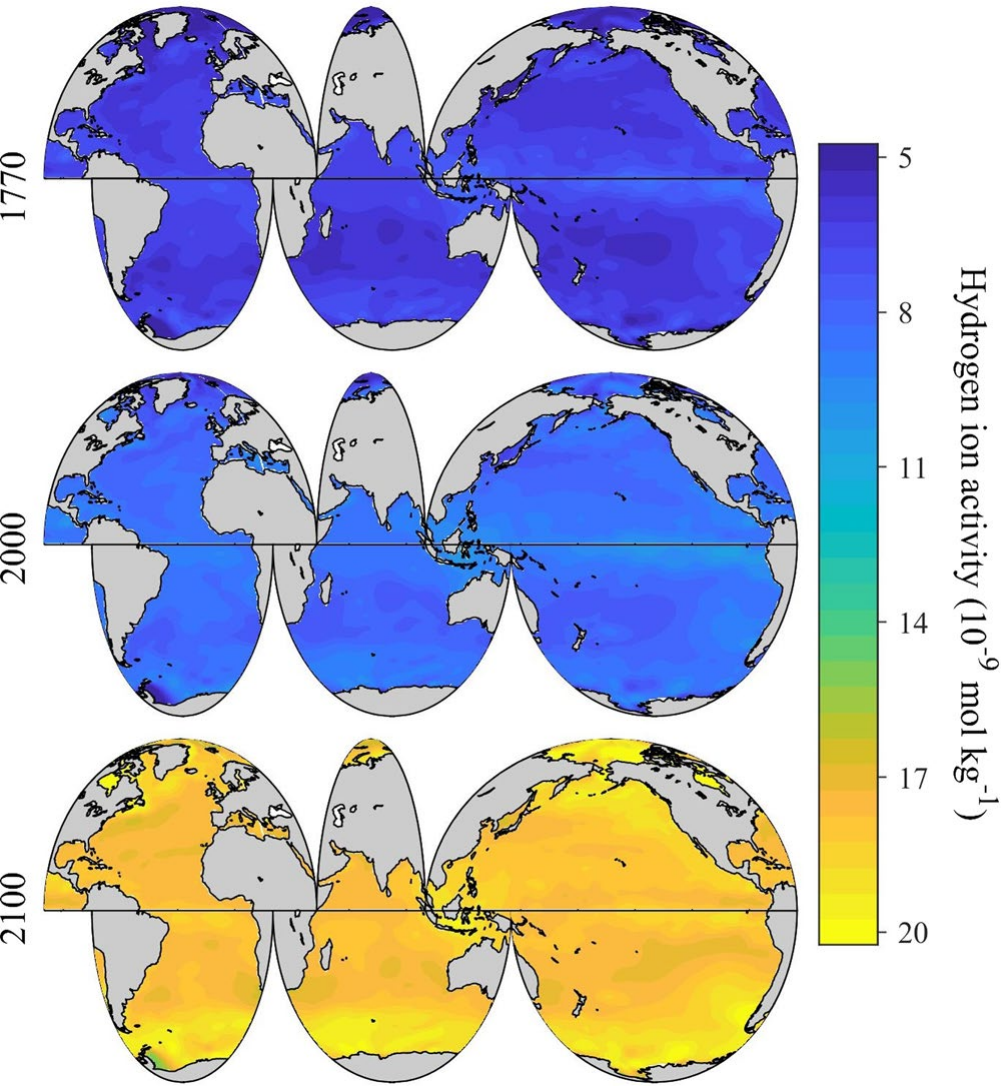
Spatial distribution of global surface ocean acidity (hydrogen ion activity, annually averaged) in past (1770), present (2000) and future (2100) under the IPCC RCP8.5 Scenario.

**Figure 8:**
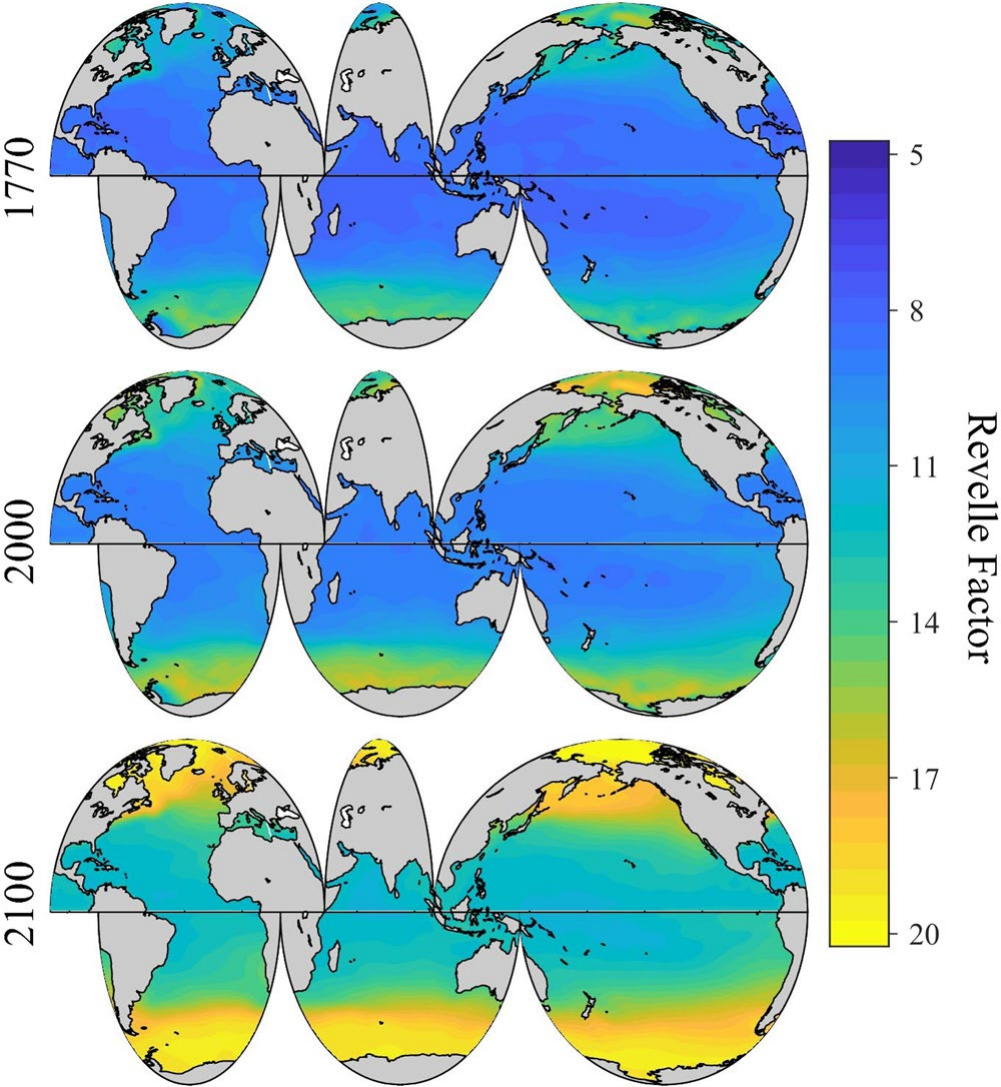
Spatial distribution of global surface ocean Revelle Factor (annually averaged) in past (1770), present (2000) and future (2100) under the IPCC RCP8.5 scenario.

Over a decade of CO_2_ emissions since 2005 show that the Representative Concentrations Pathway (RCP2.6) scenario is unlikely to adequately represent the future atmospheric CO_2_ level. Consequently, here we focus discussion instead on the RCP4.5 and RCP8.5 scenarios, as these are now the plausible low-end and high-end concentration pathways. Under the RCP 8.5 “business-as-usual” scenario, surface ocean pH_T_ changes at about –0.02 units decade^−1^ (~6% [H^+^] decade^−1^) at the beginning of this century, close to what was observed by Lauvset *et al*.^27^, and gradually accelerates to an average of about −0.04 units decade^−1^ (~10% [H^+^] decade^−1^) towards the end of the century (Fig. 5). From 2000 to 2100, the globally and annually-averaged surface ocean pH_T_ decreases by an average of about ~0.33 ± 0.04 (spatial variability) units (Figs. 5a, 6, and Supplementary Fig. S5), consistent with research based on ensembles of Earth System Models^20,21^. This is equivalent to an average hydrogen ion increase of ~114% (Figs. 5b, 7, and Supplementary Fig. S6), and slightly greater than the entire modern surface ocean pH range (Fig. 1a). The globally and annually-averaged Revelle Factor increases by 3.7 ± 0.9 (~34%) under the RCP8.5 scenario over the same period (Figs. 4b, 8, and Supplementary Fig. S7).

The magnitude of pH_T_ change from 2000 to 2100 (∆pH_T_) shows a clear latitudinal pattern with relatively small changes in the equatorial upwelling regions and larger changes in the Arctic and Southern Oceans (Supplementary Fig. S8). The latitudinal gradient in ∆pH_T_ is mainly due to the regionally varying buffer capacity (Fig. 4a) combined with the magnitude of the projected seawater *p*CO_2_ change (Supplementary Fig. S8) from 2000 to 2100. Upwelling waters tend to have the lowest concentration of anthropogenic carbon, such that anthropogenic pH change is transiently slowed down by continuous upwelling. The overall results are that surface ocean pH decreases slower in areas with lower pH, e.g. the equatorial upwelling regions. Therefore, global surface ocean pH_T_ is becoming increasingly more homogeneous with time (Fig. 6 and Supplementary Fig. S5).

Under the RCP4.5 scenario, surface ocean pH_T_ decreases at a much slower rate (Fig. 5a and Supplementary Fig. S9). In 2100, pH_T_ is ~0.13 ± 0.01 (spatial variability) units lower than in 2000, equivalent to a hydrogen ion increase of ~36% (Fig. 5b and Supplementary Fig. S10), comparable to the change from pre-industrial times until today. The globally and annually-averaged Revelle Factor would increase by 1.5 ± 0.7 (~14%) under the RCP4.5 scenario (Fig. 4b and Supplementary Fig. S11).

### Summary and conclusions

This study combines observation-based pH distribution (~23 million observations from SOCATv6) with temporal trends at individual locations of the global ocean from a robust Earth System Model to provide a high-resolution regionally varying view of global surface ocean pH and Revelle Factor in all 12 months of the year from 1770 to 2100. As a result of the tremendously enhanced observational data coverage from the SOCATv6, the climatology of this study is likely to provide improved regional OA trajectories than the model output could alone, and therefore, will help guide the regional OA adaptation strategies.

We find that SST imposes two large but cancelling effects on surface ocean pH: (a) the effects of SST on chemical speciation of the carbonate system; and (b) the air-sea exchange of CO_2_ and the subsequent DIC/TA ratio of the water. These two processes act in concert for aragonite saturation state but oppose each other for pH. As a result, surface ocean pH shows little latitudinal variation, while aragonite saturation state is markedly lower in the colder polar regions than in the warmer subtropical and tropical regions. The spatial variation of surface ocean pH is instead mainly attributable to air-sea CO_2_ disequilibria driven by temperature changes and upwelling on regional scales.

From 1770 to 2000, the global average surface ocean pH_T_ decreased by ~0.11 ± 0.03 (spatial variability) units. Under the IPCC RCP8.5 “business-as-usual” scenario, the globally and annually-averaged surface ocean pH_T_ would decrease by an additional ~0.33 ± 0.04 units (~114% increase in hydrogen ion) and the ocean’s buffer capacity would decrease by an average ~34% from 2000 to 2100. The rapid decrease in buffer capacity suggests that while the ocean will likely to continue to take up more CO_2_ in the future due to the ever increasing atmospheric CO_2_ concentration, the ocean’s role in absorbing anthropogenic CO_2_ will gradually diminish into the future, and the subsequent chemistry change in the ocean will accelerate.

## Methods

Seawater CO_2_ chemistry data needed for the pH and Revelle Factor calculations were extracted from the 6^th^ version of the Surface Ocean CO_2_ Atlas (SOCATv6, 1991–2018, ~23 million observations)^23^. Data without quality control flags of A or B (uncertainty of fugacity of carbon dioxide, *f*CO_2_ < 2 µatm) were omitted. The fugacity of carbon dioxide is an effective partial pressure of carbon dioxide with its non-ideality corrected. The difference between *p*CO_2_ and *f*CO_2_ is usually <0.5%. Silicate and phosphate values for all SOCATv6 stations were extracted from the gridded Global Ocean Data Analysis Project version 2 (GLODAPv2) climatologies^32^. Total alkalinity (TA) was then estimated based on salinity, sea surface temperature (SST), and silicate using the updated locally interpolated alkalinity regression (LIARv2) method^24^.

pH on the total hydrogen scale (pH_T_) and Revelle Factor were calculated from *in-situ* temperature, salinity, *f*CO_2_, total alkalinity (TA), silicate and phosphate using the dissociation constants for carbonic acid of Lueker *et al*.^33^, potassium bisulfate (KHSO_4_^–^) of Dickson^34^, boric acid of Dickson^35^, and with the total borate concentration equations of Uppstrom^36^ as recommended by Orr *et al*.^37^ using the MATLAB version^38^ of the CO2SYS program^39^. Uncertainties of this approach were estimated using the CO2SYS errors program^37^ to be around 0.01 pH units, with default errors for carbonate and borate system constants, and assuming uncertainties for SST, salinity, TA, and *f*CO_2_ are 0.01, 0.1, 18 µmol kg^−1^ (i.e., twice LIARv2 uncertainty estimates for these surface data^24^), and 2 µatm, respectively.

For comparison purposes, global surface ocean pH_T_ was also calculated from GLODAPv2 (1972–2012, ~13,000 observations)^26^. The GLODAPv2 data were extracted if they met these three criteria: (a) containing both dissolved inorganic carbon (DIC) and TA, (b) the World Ocean Circulation Experiment (WOCE) quality control flags for temperature, salinity, DIC, and TA = 2 (2 - good), (c) sampling depth <=20 meters. In the end, a total of 12,812 stations were used for this analysis. Global surface ocean pH_T_ was calculated using the CO2SYS program as before except that DIC is used in place of *f*CO_2_. Uncertainties of the GLODAPv2 approach were estimated to be around 0.02 pH units, assuming uncertainties for temperature, salinity, TA, and DIC are 0.01, 0.02, 6 µmol kg^−1^ and 4 µmol kg^−1^, respectively. For both calculations of pH, there are substantial uncertainties (0.01–0.02) owing to carbonate system intercomparability, in that different values of pH can be obtained using different carbonate constant sets. Therefore, neither calculation of pH should be considered more accurate than 0.01 to 0.02.

The calculated pH_T_ and Revelle Factor were adjusted from their sampling year to 2000 by assuming: (a) sea surface *p*CO_2_ increases at the same rate as atmospheric mole fraction of carbon dioxide (xCO_2_) as documented by the Intergovernmental Panel on Climate Change (IPCC) Fifth Assessment Report 5 (AR5)^40^, (b) SST increases at the rate described by 5^th^ version of NOAA’s Extended Reconstructed Sea Surface Temperature (ERSSTv5)^41^, and (c) salinity and TA remain constant.

Surface ocean pH_T_ and Revelle Factor were further adjusted from their sampling month to all 12 months of 2000 by assuming: (a) sea surface *p*CO_2_ follows the same annual cycle as documented by the Lamont-Doherty Earth Observatory (LDEO) climatology^19^, (b) sea surface temperature (SST) in all months of 2000 can be approximated by the 1995–2004 average monthly SST climatology from the World Ocean Atlas (WOA)^13^, and (c) salinity and TA remain constant.

Spatial mapping was conducted using a MATLAB version (Divand Software) of the Data-Interpolating Variational Analysis (DIVA)^42^. Correlation lengths of 42° Longitude × 21° Latitude and a Signal to Noise ratio (SN) of 3.0 were chosen to minimize disagreements between pH gridded based on SOCATv6 calculations and that based on GLODAPv2 calculations. The average uncertainty from the DIVA gridding in the Atlantic, the Pacific, and the Indian Ocean (between 60°S and 60°N) is 0.01 ± 0.01, (spatial variability), 0.01 ± 0.02 and 0.02 ± 0.03 pH units, respectively (Supplementary Fig. S12). The value in the Southern Ocean (south of 60°S) is 0.02 ± 0.04, and it is largest in the Arctic (north of 70°N) at 0.06 ± 0.17. Overall, the uncertainty is much larger in the southern hemisphere than in the northern hemisphere due to observation sparsity (Supplementary Fig. S12). For example, the mean uncertainty from 20°S to 60°S in the South Pacific is 0.02 ± 0.02 (spatial variability), about 7-fold greater than the average uncertainty from 20°N to 60°N in the North Pacific, or 0.003 ± 0.002 (Supplementary Fig. S12). Also, note that the uncertainty from the DIVA gridding is dependent on the choice of correlation lengths and SN so that different choices from those used here will yield different uncertainties.

Surface ocean pH_T_ and Revelle Factor in all 12 months for all decades from 2010 to 2100 under the four IPCC RCP scenarios (RCP2.6, RCP4.5, RCP6.0, and RCP8.5) were simulated by assuming sea surface *p*CO_2_ and SST increase at the rate simulated by the Geophysical Fluid Dynamics Laboratory (GFDL) ESM2M model run^20^. This approach replaces the traditional assumption that surface *p*CO_2_ will track atmospheric xCO_2_ everywhere in the global ocean, and allows for the possibility that parts of the surface ocean will lag behind or exceed atmospheric xCO_2_ changes due to (for example) circulation changes or incomplete air-sea equilibration.

Because sea surface *p*CO_2_ was not available as one of the GFDL-ESM2M outputs, the temporal change of *p*CO_2_ was derived based on the changes of atmospheric xCO_2_ and delta air-sea *p*CO_2_ (Eq. 2):

2 $$p{{\rm{CO}}}_{2}({\rm{year}})-p{{\rm{CO}}}_{2}(2000)=[{\rm{atm}}\,{{\rm{xCO}}}_{2}({\rm{year}})-{\rm{atm}}\,{{\rm{xCO}}}_{2}(2000)]+[\Delta p{{\rm{CO}}}_{2}({\rm{year}})-\Delta p{{\rm{CO}}}_{2}(2000)]$$

where *p*CO_2_ is the partial pressure of carbon dioxide in the ocean, atm xCO_2_ is the mole fraction of carbon dioxide in the atmosphere, and ∆*p*CO_2_ is the difference between atmospheric and oceanic partial pressure of CO_2_ (positive meaning ocean >atmosphere). Year inside the parenthesis can be 2010, 2020, … 2100. Atmospheric xCO_2_ and *p*CO_2_ are two different concepts and they vary in value, but their respective temporal changes in the surface ocean can be assumed to be reasonably close to each other.

Specifically, data for atmospheric xCO_2_ (“CO_2_”) and delta air-sea *p*CO_2_ (“∆*p*CO_2_”) were downloaded from the “Amon” and “Omon” tables of the GFDL-ESM2M model results for all four RCPs. They were subtracted by their corresponding pre-industrial control (pi-control) values and then averaged over 10 years. For example, data from January 1, 2005 to December 31, 2014 were used to calculate the atmospheric xCO_2_ and ∆*p*CO_2_ values in 2010. Then the seawater *p*CO_2_ change from 2000 to 2010 at a certain location was calculated according to Eq. 2. We similarly calculated the seawater *p*CO_2_ change relative to 2000 for all decades through 2100.

The temporal change of SST in all decades of the 21^st^ century relative to 2000 was derived from the GFDL-ESM2M model outputs as well. Temperature of surface (“TOS”) and its “pi-Control” were downloaded from the “Omon” table of the GFDL-ESM2M results. Once we had the projected seawater *p*CO_2_ and SST values, by assuming constant salinity and TA, we were able to calculate pH at each location of the global ocean in all months for all decades of the 21st Century under the four RCP scenarios. The assumption of a constant TA could bring in a maximum pH bias of 0.004 pH units, much smaller than the pH calculation uncertainty of 0.01 pH units^43^.

Similarly, the historical surface ocean pH and Revelle Factor from 1870 to 1990 were calculated using the “esmHistorical” data from the GFDL-ESM2M model results. Because the GFDL-ESM2M model outputs start from January 1, 1861 and at least 10 years’ worth of data were needed to do a temporal average to estimate surface ocean pH in any decade, the earliest decade we could calculate pH for started in 1870. However, by assuming surface ocean *p*CO_2_ changes at the same rate as the atmospheric xCO_2_^40^ everywhere in the global ocean, and SST stays the same from 1770 to 1860, we were able to calculate surface ocean pH in all months for all decades from 1770 to 1860 as well. Considering atmospheric xCO_2_ only changed 8.9 µatm from 1770 to 1870 (equivalent to a pH change of ~0.01 units), and the pH calculation is insensitive to temperature, the above approximation is unlikely to result in significant uncertainties in pH values over that timeframe.

The influences of the two temperature processes on surface ocean pH (chemical speciation vs. gas exchange) were quantified by examining seawater at the average global SST, salinity, DIC, and TA of 18.35 °C, 34.87, 2020 µmol kg^−1^, 2306 µmol kg^–1^ respectively^26^. The impact of the first process, i.e., the temperature dependence of the chemical speciation of seawater CO_2_ chemistry species, was isolated by assuming constant TA and DIC and varying temperatures of [0 °C:5 °C:30 °C], reflecting variations from the poles to the equator. Hydrogen ion (H^+^) generation caused by the dissociation of HCO_3_^−^ can be estimated from the change of carbonate ion concentration ([CO_3_^2−^]). Similarly, H^+^ generation caused by the dissociation of H_2_O can be estimated from the change of hydroxide ion concentration ([OH^−^]). H^+^ consumption caused by borate can be estimated from the change of borate alkalinity [B(OH)_4_^−^], and that caused by its reaction with HCO_3_^−^ can be estimated from the change of aqueous carbon dioxide concentration ([CO_2_*]).

The impact of the second process, i.e., temperature-driven air-sea CO_2_ exchange was isolated by assuming fixed TA and *p*CO_2_ from the equator to the poles, varying input temperature to [0 °C:5 °C:30 °C] from the poles to the equator, while keeping output temperature constant. The combined influences of both processes can be estimated with a fixed TA and *p*CO_2_, while setting both input and output temperatures to [0 °C:5 °C:30 °C].

## Supplementary information

Supplementary materials for Surface ocean pH and buffer capacity: past, present and future

## Data Availability

Data used for this analysis are from the 6^th^ version of the Surface Ocean CO_2_ Atlas (SOCATv6), and the 2^nd^ version of the Global Ocean Data Analysis Project (GLODAPv2) data product, which are available at National Oceanic and Atmospheric Administration (NOAA) National Centers for Environmental Information (NCEI) [DOI:10.7289/V51Z42R8 (SOCATv6), and DOI:10.7289/V5KW5D97 (GLODAPv2)]. The data products of this study, i.e., the climatological distributions of global surface ocean pH, acidity, and Revelle Factor in all 12 months of the year from 1770 to 2100 (gridded data in netCDF format), as well as Powerpoint slides and animations for presentation, are available through NOAA/NCEI (DOI: 10.25921/kgqr-9h49). Please note that both SOCATv6 and GLODAPv2 are primarily open ocean based products. As a result, uncertainties in the coastal ocean could be very large due to the lack of data coverage and the subsequent gridding artifacts. The scientific results and conclusions, as well as any views or opinions expressed herein, are those of the authors and do not necessarily reflect the views of NOAA or the Department of Commerce.|
