# Supplementary material for: Surface ocean pH and buffer capacity: past, present and future
Source: Sci Rep. 2019 Dec 9;9:18624. doi: 10.1038/s41598-019-55039-4 (PMC6901524; doi:10.1038/s41598-019-55039-4)
Supplement: Supplementary file 1 — Supplementary materials for Surface ocean pH and buffer capacity: past, present and future [file 41598_2019_55039_MOESM1_ESM.pdf]

Supplementary materials for

Surface ocean pH and buffer capacity: past, present and future

Li-Qing Jiang[1,2,*], Brendan R. Carter[3,4], Richard A. Feely[4], Siv K. Lauvset[5,6], and Are Olsen[6]

[1] Earth System Science Interdisciplinary Center, University of Maryland, College Park, Maryland, USA.

[2] National Centers for Environmental Information, National Oceanic and Atmospheric Administration, Silver Spring, Maryland, USA.

[3] Joint Institute for the Study of the Atmosphere and Ocean, University of Washington, Seattle, Washington, USA.

[4] Pacific Marine Environmental Laboratory, National Oceanic and Atmospheric Administration, Seattle, Washington, USA.

[5] NORCE Norwegian Research Centre, Bjerknes Centre for Climate Research, Bergen, Norway.

[6] Geophysical Institute, University of Bergen and Bjerknes Centre for Climate Research, Bergen, Norway.

[*] Corresponding author: L.-Q. Jiang, Earth System Science Interdisciplinary Center, University of Maryland, 5825 University Research Court, College Park, MD 20740, USA. (Liqing.Jiang@noaa.gov).

[The scientific results and conclusions, as well as any views or opinions expressed herein, are those of the authors and do not necessarily reflect the views of NOAA or the Department of Commerce.]

Table S1. The reaction of carbonic system species to a temperature change of 20 to 25°C in a closed system with constant dissolved inorganic carbon (DIC) and total alkalinity (TA). The calculation is based on an imaginary seawater with SST, salinity, DIC, and TA of 18.35°C, 34.87, 2020 μmol kg^{-1}, 2306 μmol kg^{-1}, respectively. The rightmost column shows how fugacity of carbon dioxide (fCO_2) would change if all of the hydrogen ion (H^+) change were used to react with bicarbonate (HCO_3^-) to form aqueous CO_2 (CO_2*, a combination of both dissolved CO_2 and carbonic acid, or H_2CO_3) (or reverse) and the CO_2 solubility remained constant at the 20°C level. Please note that in this example, we used a temperature change of 20 to 25°C. Their values could be very different at other temperature change intervals. This is especially true for the dissociation of water (H_2O).

	**Processes**	**H^+ change (μmol/kg)**	**fCO_2 change (μatm)**
Generating H^+	HCO_3^- dissociation	+1.99	61
	H_2O dissociation	+1.55	48
Consuming H^+	Reacting with $B(OH)_4^-$ to form $B(OH)_3$	−2.72	−84
	Reacting with HCO_3^- to form CO_2*	−0.82	**25**
H^+ surplus	Staying as free H^+ or forming H_3O^+ to lower pH	**0.0015**	−0.05
fCO_2 change due to K_0 change	K_0 decreases with increasing temperature	N/A	**53**
fCO_2 change due to changes in both [CO_2*] and K_0	N/A	N/A	78

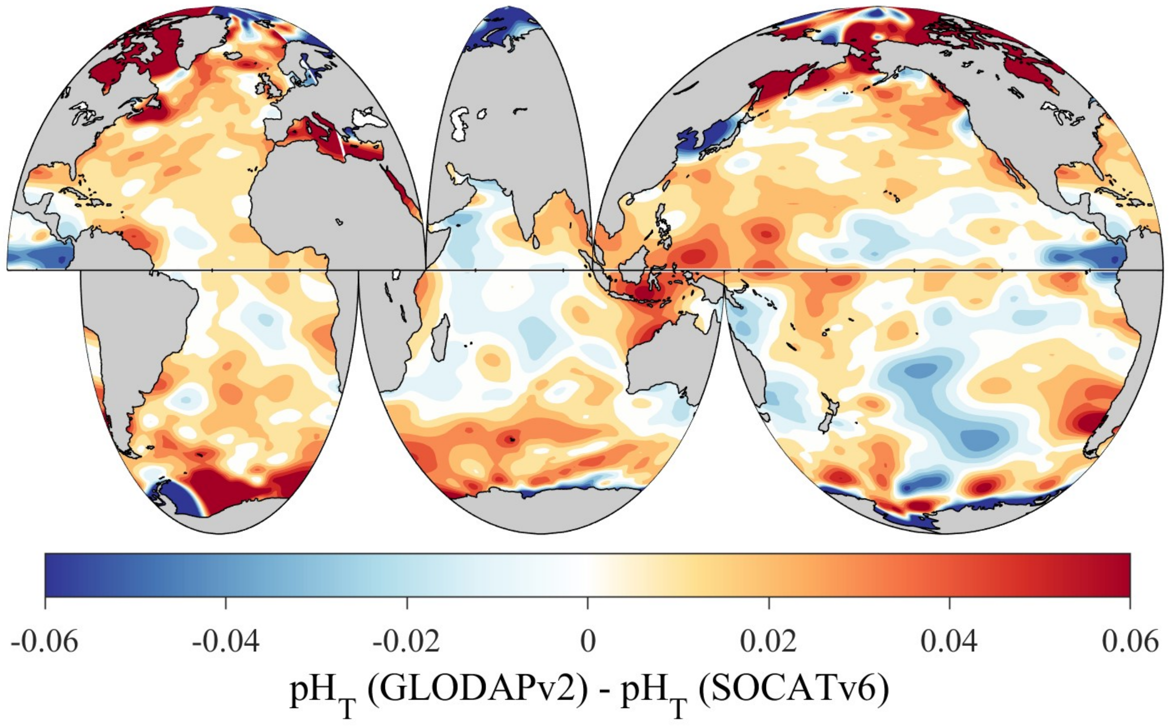

Figure S1. The difference between surface pH_T based on the 2nd version of the Global Ocean Data Analysis Project (GLODAPv2) and that of the 6th version of the Surface Ocean CO_2 Atlas (SOCATv6).

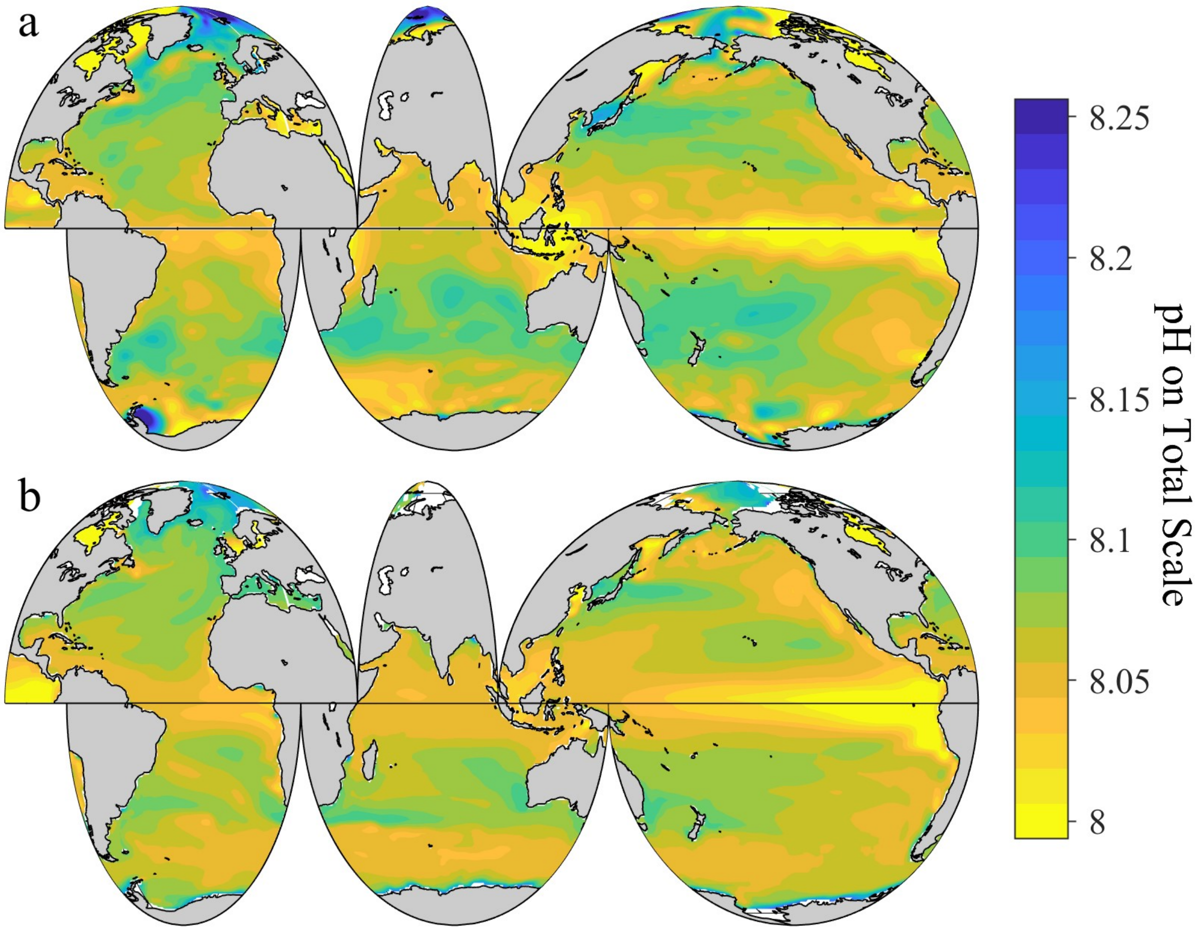

Figure S2. Climatological distribution of global surface ocean pH on the total hydrogen scale (pH_T, annually averaged) at *in-situ* temperature and adjusted for the year 2000. Panel a, the global surface ocean pH_T based on the 6th version of the Surface Ocean CO_2 Atlas (1991 to 2018, ~23 million observations). Panel b, the global surface ocean pH_T extracted from the Geophysical Fluid Dynamics Laboratory (GFDL)'s ESM2M Model.

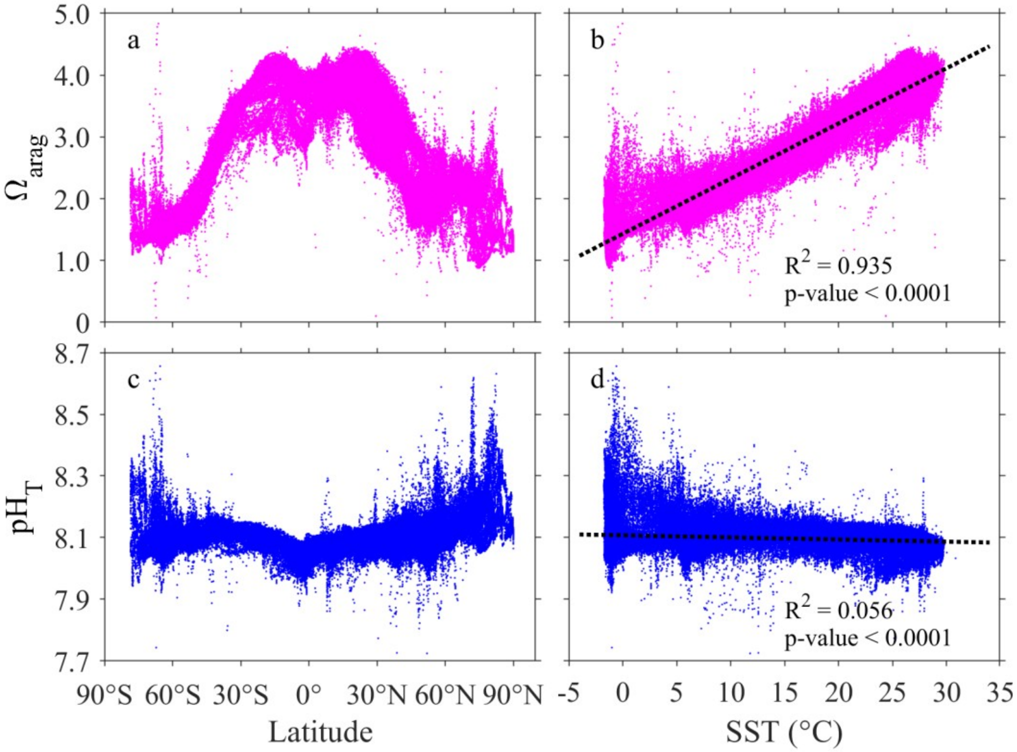

Figure S3. Plots of surface ocean aragonite saturation state (Ω_{arag}) and pH on the total hydrogen scale (pH_T) against both latitude and sea surface temperature (SST). Panel **a,** plot of Ω_{arag} against latitude. Panel **b,** plot of Ω_{arag} against SST. Panel **c,** plot of pH_T against latitude. Panel **d**, plot of pH_T against SST.

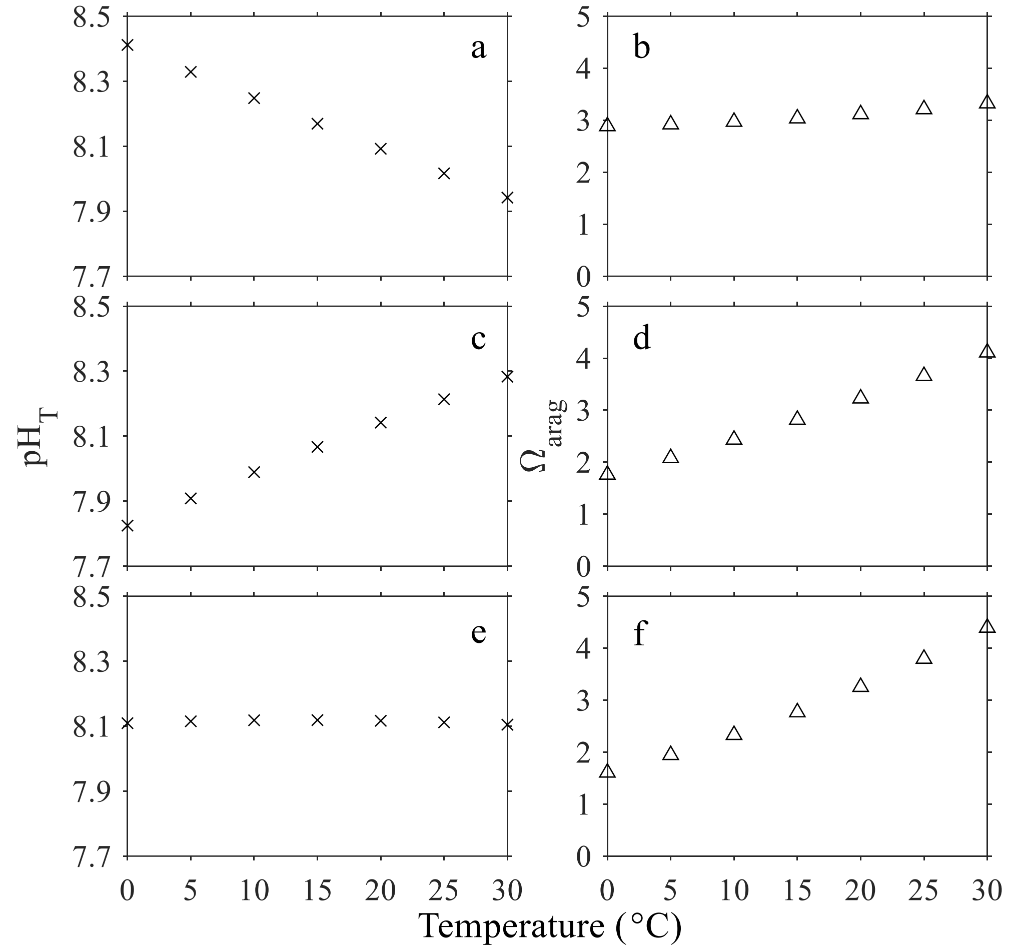

Figure S4. Plots of pH on the total hydrogen scale (pH_T), and aragonite saturation state (Ω_{arag}) under the two aspects of temperature influences against water temperature. Panels **a** and **b,** plots of pH_T and Ω_{arag} that are calculated by assuming constant TA and DIC but varying temperature (from 0 °C at the poles to 30 °C at the Equator). Panels **c** and **d,** plots of pH_T and Ω_{arag} that are calculated by assuming constant TA and pCO_2, varying input temperature, but a constant output temperature. Panels **e** and **f**, plots of pH_T and Ω_{arag} that were calculated by assuming constant TA and pCO_2, varying both input and output temperature.

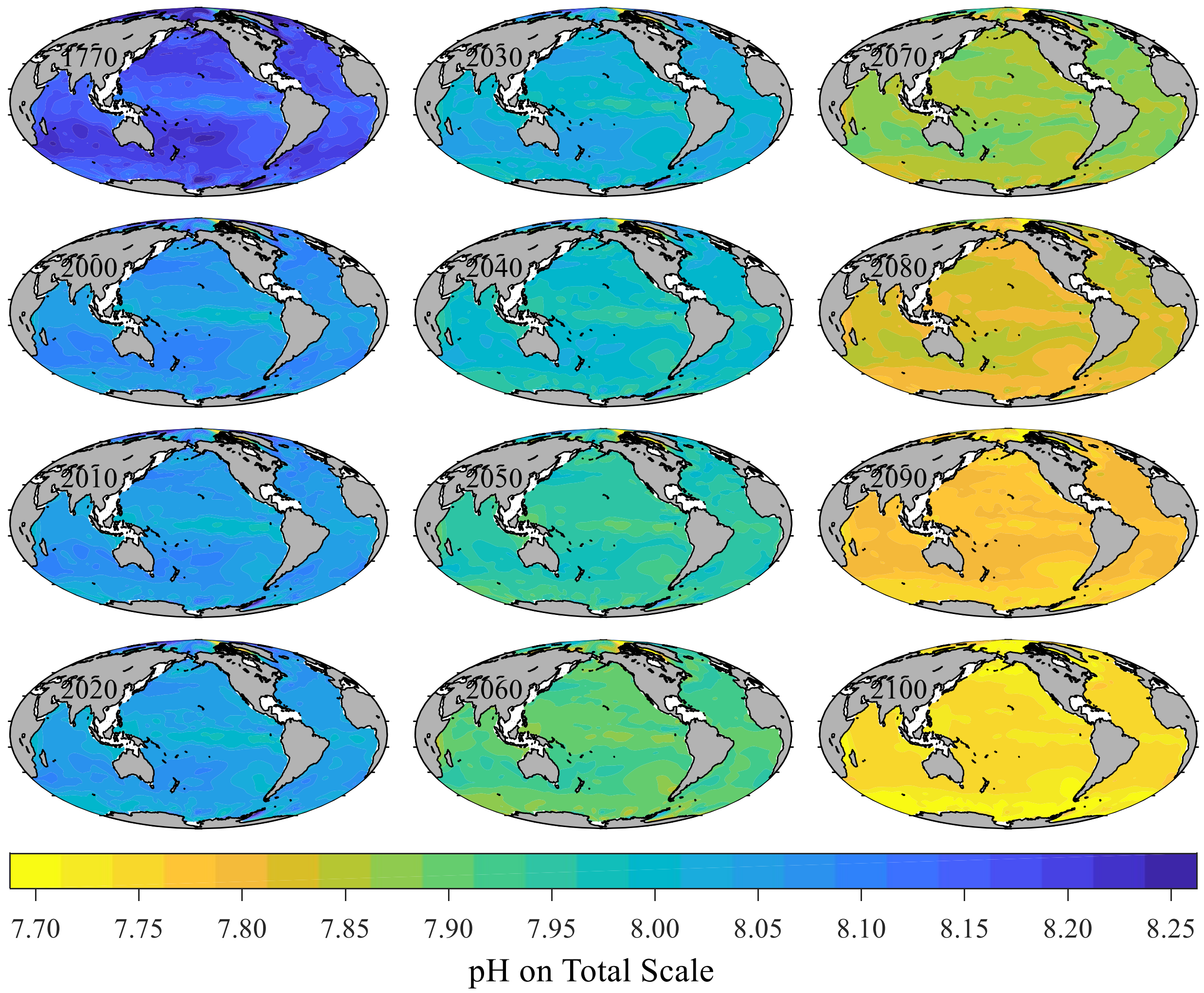

Figure S5. Surface water pH on total hydrogen scale (pH_T, annually averaged) in 1770, 2000, and all decades of the 21st Century under the Intergovernmental Panel on Climate Change (IPCC) Representative Concentrations Pathways (RCP8.5) Scenario.

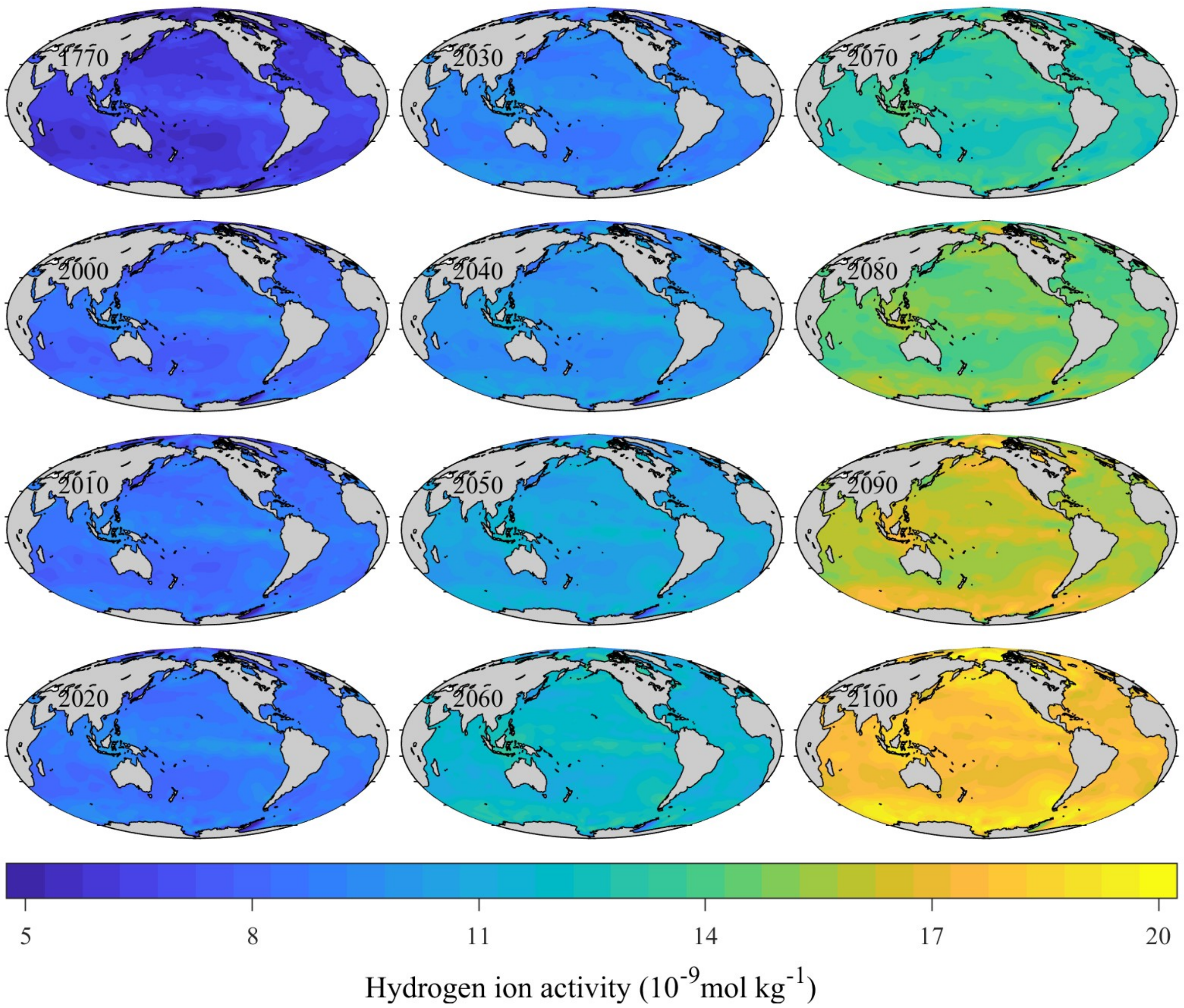

Figure S6. Surface water hydrogen ion activity ([H^+], annually averaged, unit: 10^{-9} mol kg^{-1},) in 1770, 2000, and all decades of the 21st Century under the IPCC RCP8.5 Scenario.

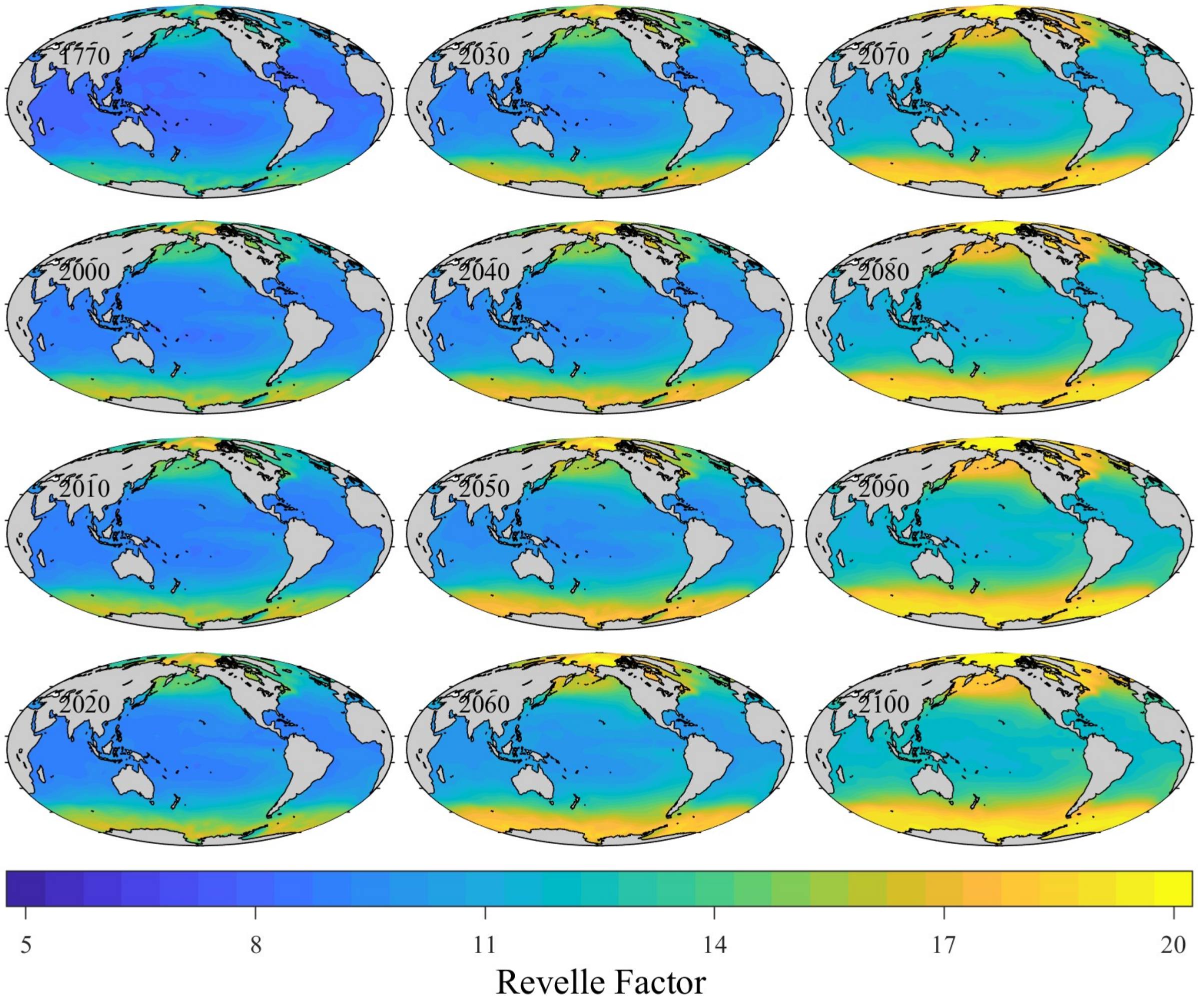

Figure S7. Surface water Revelle Factor (annually averaged) in 1770, 2000, and all decades of the 21st Century under the IPCC RCP8.5 Scenario. Revelle Factor is a measure of the ocean's buffer capacity (the higher the Revelle Factor, the lower the ocean's buffer capacity).

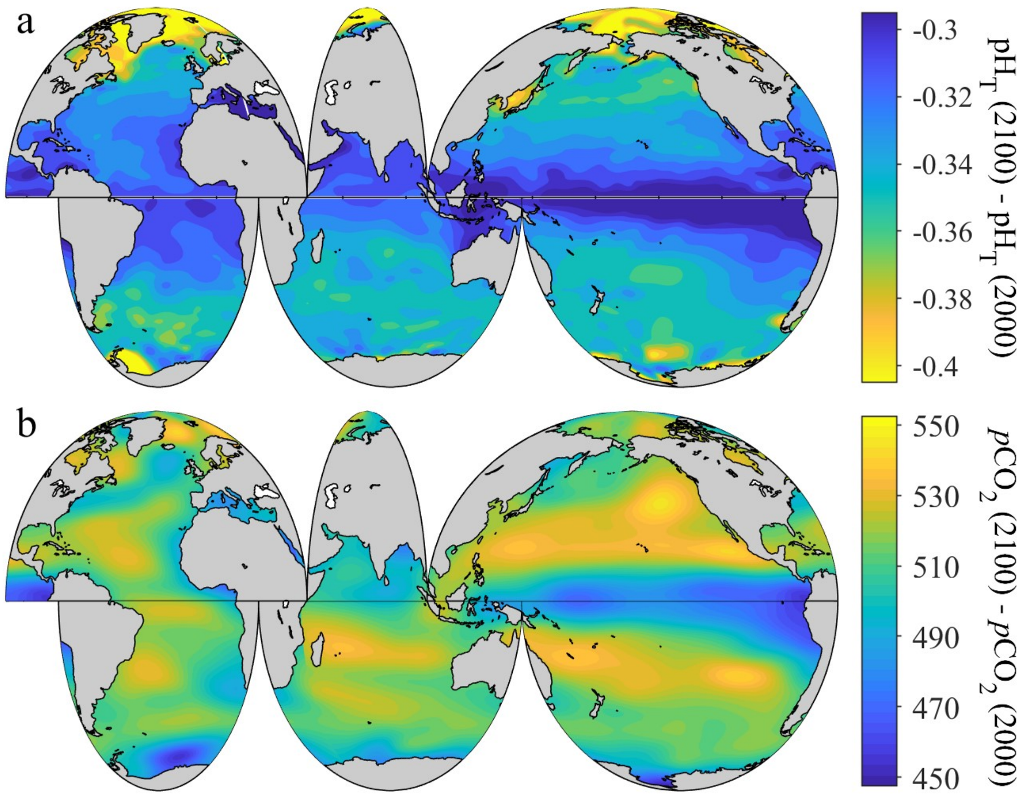

Figure S8. Long-term changes of surface ocean pH on the total hydrogen scale (pH_T) and partial pressure of carbon dioxide (pCO_2) from 2000 to 2100. Panel a, the global surface ocean pH_T change from 2000 to 2100. Panel **b,** the global surface ocean pCO_2 change (µatm) from 2000 to 2100 under the IPCC RCP8.5 Scenario.

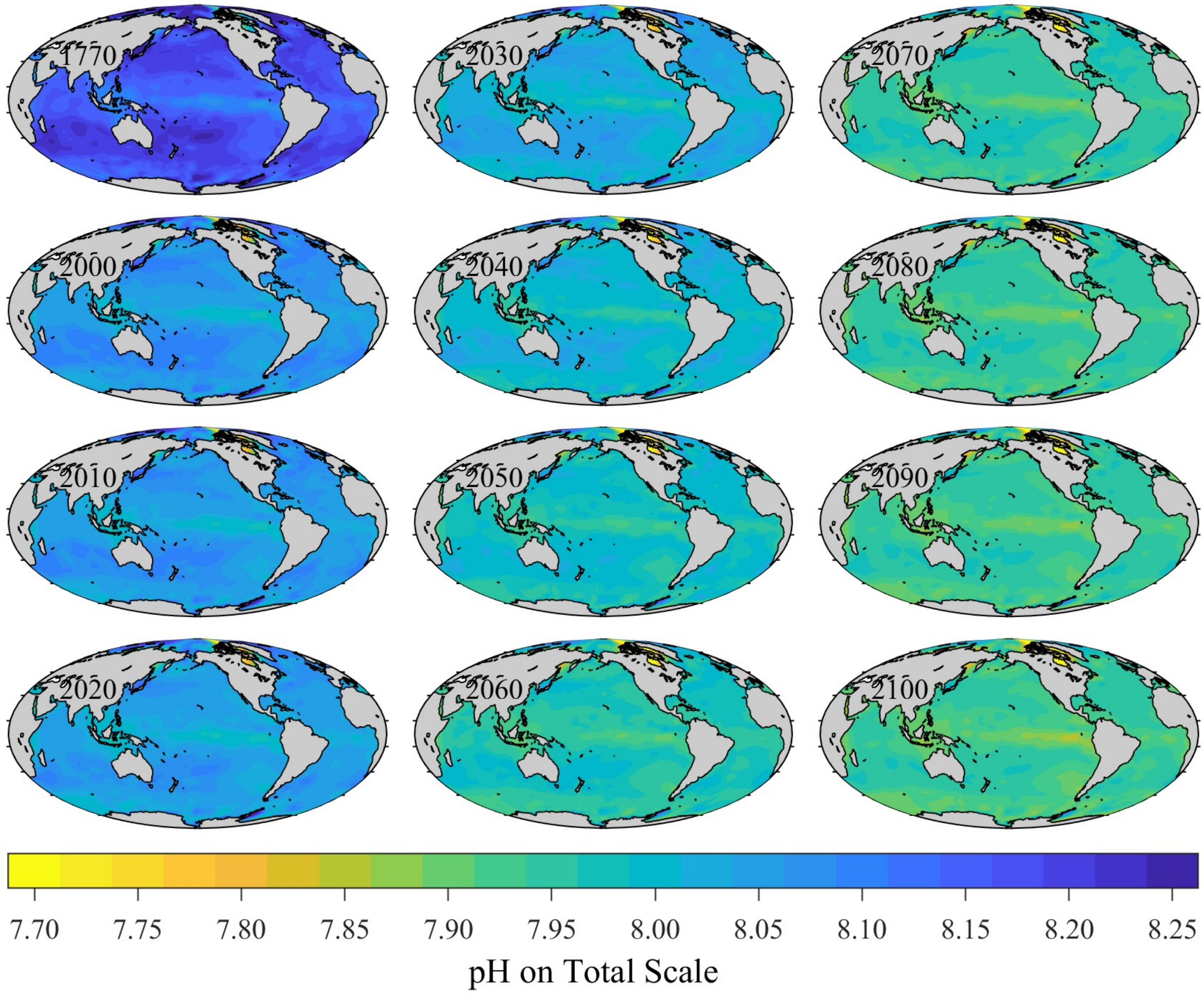

Figure S9. Surface water pH on total hydrogen scale (pH_T, annually averaged) in 1770, 2000, and all decades of the 21st Century under the IPCC RCP4.5 Scenario.

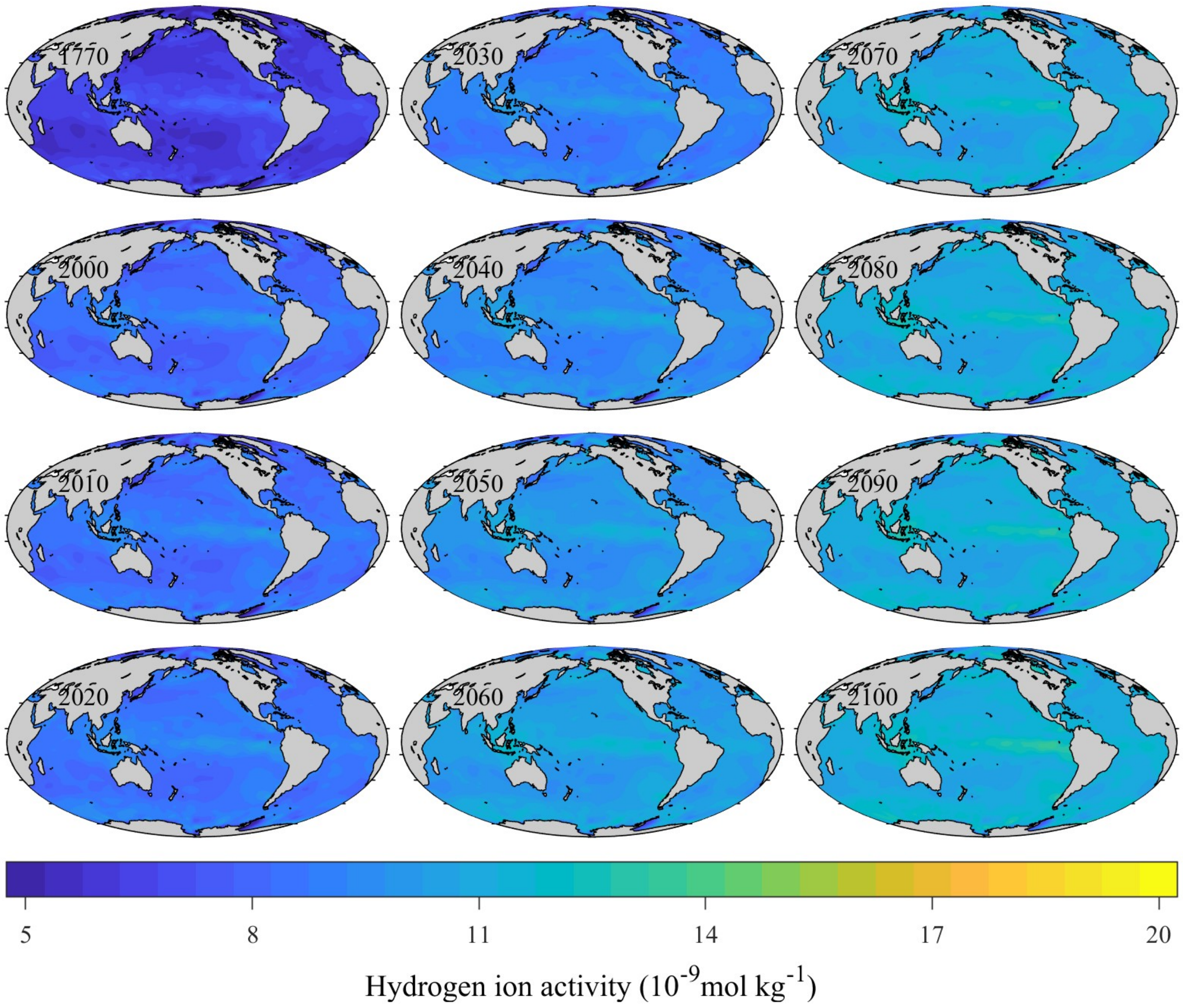

Figure S10. Surface water hydrogen ion activity ($[H^+]$, annually averaged, 10^{-9} mol kg^{-1}) in 1770, 2000, and all decades of the 21st Century under the IPCC RCP4.5 Scenario.

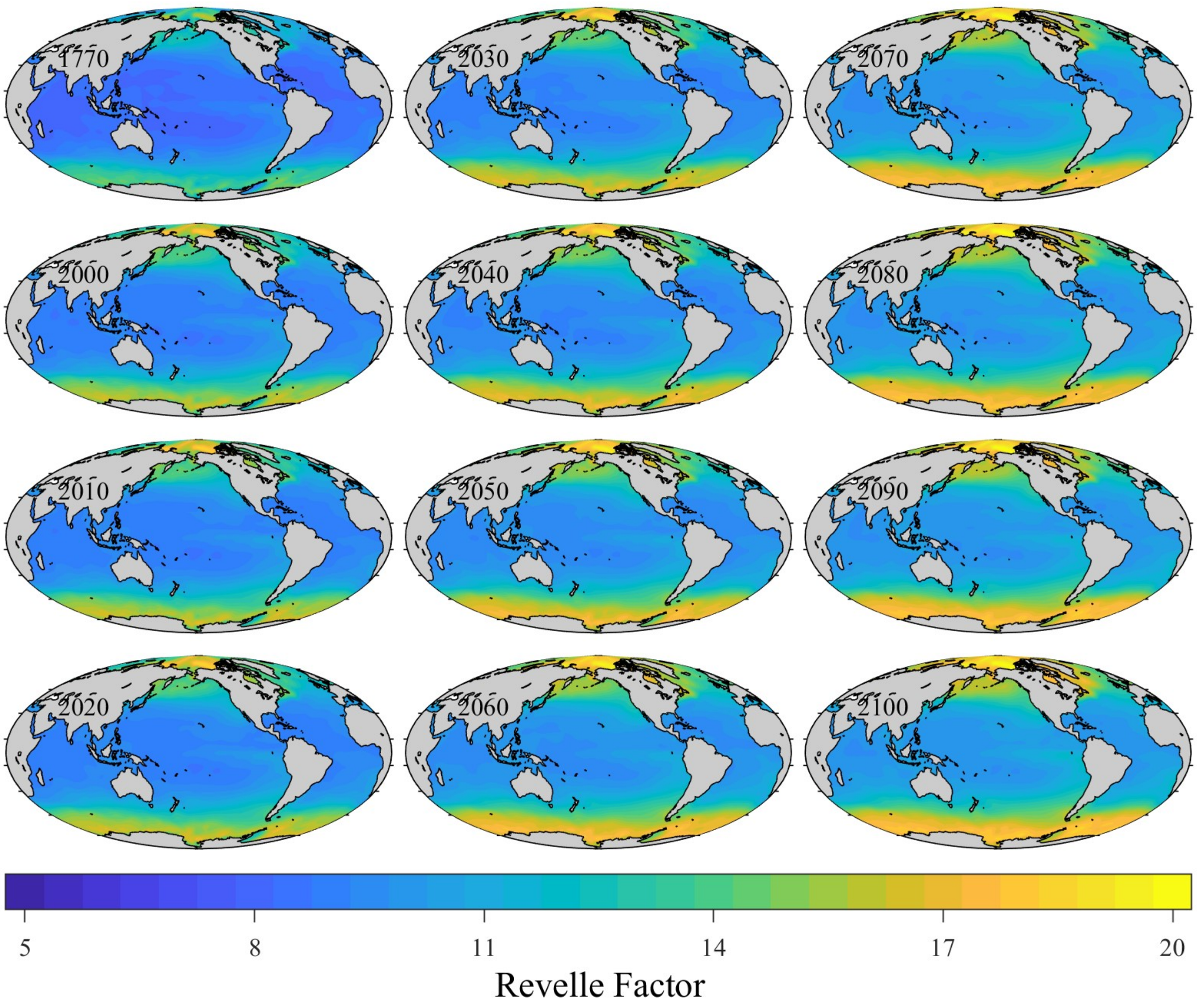

Figure S11. Surface water Revelle Factor (annually averaged) in 1770, 2000, and all decades of the 21st Century under the IPCC RCP4.5 Scenario. Revelle Factor is a measure of the ocean's buffer capacity (the higher the Revelle Factor the lower the ocean's buffer capacity).

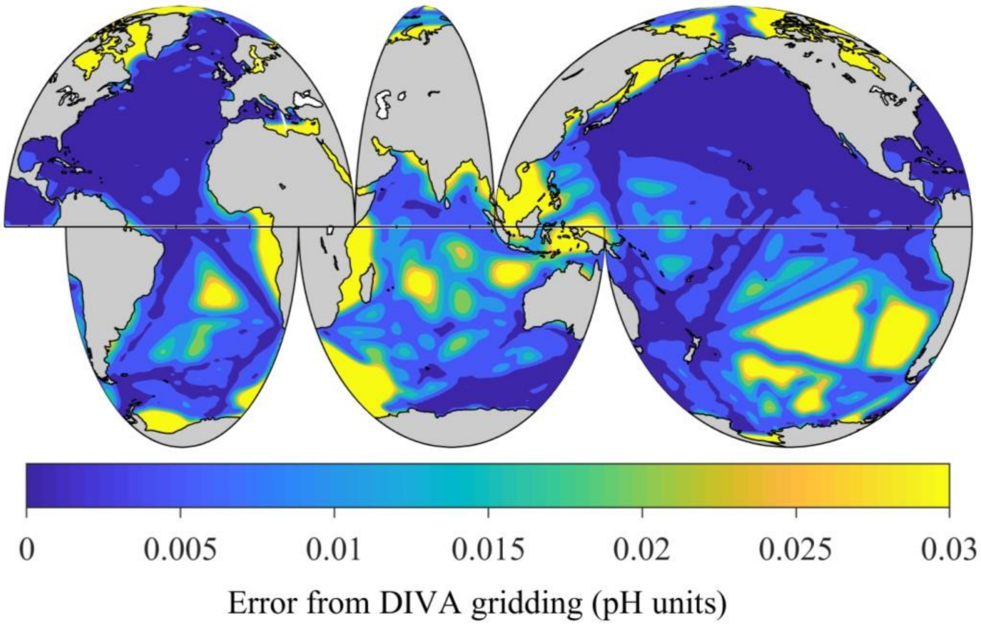

Figure S12. Surface ocean pH uncertainty from spatial gridding using the Data-Interpolating Variational Analysis (DIVA).
